# Structural Variations Identified in Patients with Autism Spectrum Disorder (ASD) in the Chinese Population: A Systematic Review of Case-Control Studies

**DOI:** 10.3390/genes15081082

**Published:** 2024-08-15

**Authors:** Sek-Ying Chair, Ka-Ming Chow, Cecilia Wai-Ling Chan, Judy Yuet-Wa Chan, Bernard Man-Hin Law, Mary Miu-Yee Waye

**Affiliations:** 1 The Nethersole School of Nursing, Faculty of Medicine, The Chinese University of Hong Kong, Hong Kong SAR, China; kmchow@cuhk.edu.hk (K.-M.C.); ceciliawlchan@cuhk.edu.hk (C.W.-L.C.); judychanyw@cuhk.edu.hk (J.Y.-W.C.); lawmanhin250@hotmail.com (B.M.-H.L.); mary-waye@cuhk.edu.hk (M.M.-Y.W.); 2 Asia-Pacific Genomic and Genetic Nursing Centre, The Nethersole School of Nursing, Faculty of Medicine, The Chinese University of Hong Kong, Hong Kong SAR, China; 3 The Croucher Laboratory for Human Genomics, The Nethersole School of Nursing, Faculty of Medicine, The Chinese University of Hong Kong, Hong Kong SAR, China

**Keywords:** autism spectrum disorder, ASD, autism, structural variation, copy number variation, CNV, genetics, Chinese, China, Han

## Abstract

Autistic spectrum disorder (ASD) is a neurodevelopmental disability characterised by the impairment of social interaction and communication ability. The alarming increase in its prevalence in children urged researchers to obtain a better understanding of the causes of this disease. Genetic factors are considered to be crucial, as ASD has a tendency to run in families. In recent years, with technological advances, the importance of structural variations (SVs) in ASD began to emerge. Most of these studies, however, focus on the Caucasian population. As a populated ethnicity, ASD shall be a significant health issue in China. This systematic review aims to summarise current case-control studies of SVs associated with ASD in the Chinese population. A list of genes identified in the nine included studies is provided. It also reveals that similar research focusing on other genetic backgrounds is demanded to manifest the disease etiology in different ethnic groups, and assist the development of accurate ethnic-oriented genetic diagnosis.

## 1. Introduction

Autism spectrum disorder (ASD) is a complex neurodevelopmental condition characterised by diverse symptoms that affect communication, behaviour, and social interactions. For a period, the prevalence of the disorder in the US was believed to be around 1 in 110 children (data of 2006), but it was found that it raised substantially to 1 out of 36 in a recent report by the Centers for Disease Control and Prevention (CDC) [1]. In China, according to an investigation in 2019, the prevalence of ASD children was 1%, which is comparable to the worldwide data, but the number was believed to be underestimated due to lack of awareness of the disorder and therefore under diagnosis [2].

To dissect the cause of ASD, studies focusing on genetic, environmental, and psychological aspects were conducted, and both genetic and environmental risk factors were identified [3,4,5]. Genetic factors are estimated to account for 40 to 80 percent of ASD risk. Most of the research on genetic factors focuses on single nucleotide polymorphisms (SNPs) of candidate genes associated with ASD [6,7]. Recent advances in genomic technologies ushered in a new era of understanding the genetic basis of ASD, highlighting the significant role of genetic variations in its etiology [8]. Among the genetic anomalies, structural variations (SVs), such as deletions, duplications, inversions, and copy number variations (CNVs), were identified as pivotal contributors to the disorder. These variations can disrupt gene functions or modify gene dosage, ultimately affecting synaptic plasticity and brain development. In the last two decades, our technology in genomic study gently transformed from sequence-based to assembly-based techniques. New methods, such as those of Oxford Nanopore Technologies (ONT) and Pacific Biosciences (PacBio), are typical examples of assembly based approaches [9]. Integration of these technologies with some evolving techniques, such as optical mapping, were shown to be powerful in the construction of genomes [10,11]. With these advancements, long sequence reads were made possible, which is critical to the discovery of SVs that commonly span from kilo to mega bases.

The research of genetic variations in ASD was extensive [12,13]. However, much of this was conducted in Caucasian populations, leaving a gap in the understanding of how ASD manifests in non-Caucasian populations, including the Chinese population, which contributes to 17% of the world’s population. From a research perspective, these differences highlight the need for a globally inclusive genetic database that encompasses diverse populations to ensure that genetic studies of ASD are universally applicable and not biased towards specific genetic ancestries. Clinically, understanding these differences is crucial for the development of diagnostic tools and therapeutic strategies that are effective across different genetic backgrounds [14]. For instance, genetic testing protocols optimised for Western populations may need adjustment to be as effective in Chinese populations due to these differences in SV prevalence and impact [15]. This line of inquiry is crucial given the genetic and environmental differences that may influence the prevalence and manifestations of ASD in different ethnic groups.

The current review aims to summarise the types of SVs reported in ASD among the Chinese population. It also discusses the genes potentially implicated in ASD. By examining the intersection of genetics and ethnicity, this review seeks to enhance the understanding of ASD’s complex etiology and pave the way for more effective interventions tailored to different populations.

## 2. Methods

This systematic review was conducted following the Preferred Reporting Items for Systematic Reviews and Meta-Analyses (PRISMA) guidelines and registered in Inplasy (INPLASY202480073) [16].

### 2.1. Search Strategy

Literature searches were completed in five Western databases, including PubMed, EMBASE, Ovid Medline, Ovid Nursing, and CINAHL, and four Chinese databases, including CNKI, Wanfang, Sinomed, and VIP. Searches in electronic databases identified studies published from inception to March 2024. Keywords used for searching included “Autism OR Autistic” AND “Structural varia* OR Transposition* OR Transposon* OR Retrotransposition* OR Retrotransposon* OR Insertion* OR Deletion* OR Indels OR Translocation* OR Inversion* OR Tandem repeat* OR Duplication* OR Copy number varia* OR Copy number polymorphism* OR Chromosomal rearrangement* OR Recombination* OR Microdeletion* OR Genomic varia*” AND “China OR Chinese OR Han”. Studies were extracted from systematic literature search following inclusion and exclusion criteria. Inclusion criteria included (i) human case-control cohort studies; (ii) ASD cohort recognised using any standard diagnostic criteria (Diagnostic and Statistical Manual of Mental Disorders or other clinical diagnosis). Exclusion criteria included: (i) studies not having a control group; (ii) non-English/Chinese publication; (iii) in vitro and/or animal studies; and (iv) abstracts, reviews, and study protocol.

### 2.2. Data Extraction

The Covidence software was used to remove duplicated articles and then screen the title and abstracts of the retrieved articles. Articles that satisfied the inclusion criteria were subsequently evaluated in full text and were assessed for their suitability for data extraction and analysis. The screening and data extraction of the articles were conducted by two authors independently. Any discrepancies regarding inclusion were resolved through discussion and consensus among the researchers involved. 

### 2.3. Quality Assessment

Risk of bias assessment of the articles was conducted by two authors independently. Discrepancies in ratings were resolved by discussion and consensus. The assessment of study quality was carried out using 8 specific items derived from the Strengthening the Reporting of Genetic Association Studies (STREGA) checklist [17]. The assessment criteria include a thorough description of several key aspects: (1) the genotyping methodology of the studies, such as laboratory methods, the centre that performed the genotyping, the method of false positive detection, indication of whether experiments were performed in one batch; (2) detailed data analysis approaches, including the number of samples successfully genotyped, the control of population stratification, and the methods to determine SVs; and (3) the results of the studies, indicating whether the genetic variations are reported for the first time. Each checklist item achieved by the studies earns one point. Items not met received a zero [18].

## 3. Results

### 3.1. Search Result

**Figure 1:**
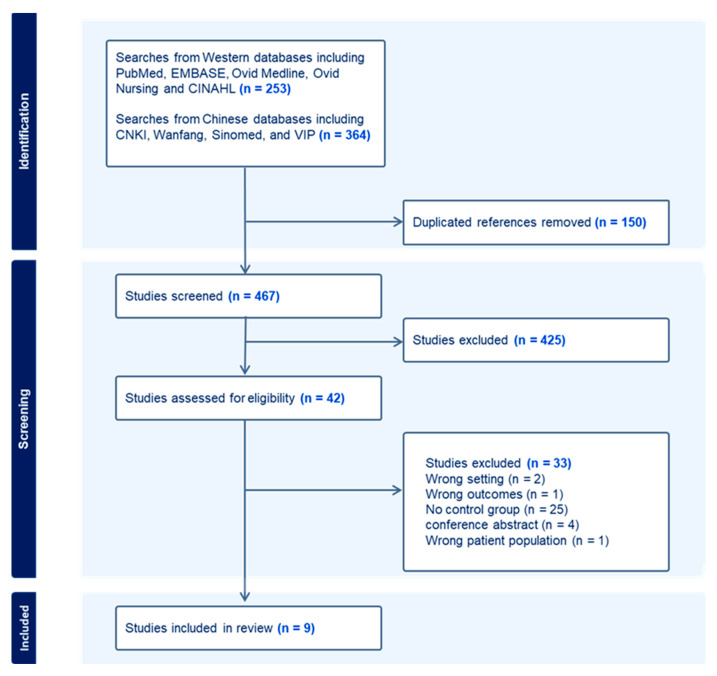
Flow diagram showing the study selection and exclusion process according to PRISMA guidelines.

As shown in the PRISMA flow diagram (Figure 1), the systematic search strategy identified 253 publications from Western databases and 364 from Chinese databases, of which 150 were duplicates. The remaining 467 studies were screened by two reviewers for relevance by title and abstract, resulting in the removal of 425 studies. Full text screening of the remaining 42 studies excluded a further 33 studies, which did not meet inclusion criteria. Most excluded studies had no control group (n = 25). Other reasons for exclusion include wrong setting (n = 2), wrong outcomes (n = 1), wrong patient population (n = 1), and not a full research publication (n = 1). Finally, a total of nine studies, which fulfilled inclusion criteria, were included and evaluated in this systematic review [19,20,21,22,23,24,25,26,27]. Eight studies were published in English and one in Chinese language. 

### 3.2. Overview of Included Studies

Table 1 characterised the studies included in this review [19,20,21,22,23,24,25,26,27]. The studies were published between 2011 and 2020. Except one study that was conducted in Taiwan, and one study that was conducted in the Hong Kong Special Administrative Region of China, all other studies were conducted in mainland China. The number of ASD subjects recruited ranged from 66 to 539. The diagnostic methods used to identify ASD individuals in the studies included the fourth edition of the Diagnostic and Statistical Manual of Mental Disorders (DSM-IV), the fifth edition of the Diagnostic and Statistical Manual of Mental Disorders (DSM-5), Autism Diagnostic Observation Schedule (ADOS), Childhood Autism Rating Scale (CARS), Autism Behaviour Checklist (ABC), and Autism Diagnostic Interview-Revised (ADI-R). 

### 3.3. Study Quality Assessment

The STREGA ratings of each included study are presented in Table 2. Overall, the quality of reporting genetic associations among the studies was low-moderate with a mean score at 3.7 and a score ranging between 2 and 6. All included studies described genotyping methods and platforms, and how the SVs were determined. None of the reports provide a hint on whether the genotyping was performed in one single batch or a few smaller batches. Only one study stated the centre at which genotyping was performed. Two studies described the methods used to control risk of false positives. About half of the studies reported the number of successful genotyping (56%) and stated whether the SVs were reported for the first time (45%). Among the nine included studies, three of them performed population stratification and two of which described how the classification was assessed. 

### 3.4. Structural Variations Identification 

Altogether, more than 70 SVs were reported in the included studies. Although the sizes of the SVs vary greatly, mainly due to the difference in technologies used, all CNVs involve deletions or duplications. Table 3 summarises the genes located at the SVs and their corresponding chromosomal banding. The SVs found could be de novo, maternal, or paternal in inheritance. The significance of some important genes implicated in autism, including *CNTNAP2*, *GABRB3*, *JARID2*, *NLGN4X*, *NRXN1*, *PARK2*, *SHANK3*, and *UBE3A*, were listed in Table 4 and reviewed in the discussion section of this review.

## 4. Discussion

In the present review, only nine studies were included. This reflects relevant research is lacking. This is not surprising because, firstly, the case-control study is more demanding, no matter the recruitment scale or experimental resources. However, case-control studies provide a more relevant comparison and effective analysis for exploring associations and risk factors. A familial study may be preferable when resources are limited. Secondly, the public awareness of ASD in China just started to emerge in the last two decades. With the development of standardised diagnostic instruments and the rise in public education of ASD in China, there shall be great potential to conduct similar research in the country considering the large population of Chinese people. Alongside the advancement of technologies in the discovery of SVs in genomics, a better understanding of the genetic factors for ASD in the Chinese population shall be attained. Nevertheless, studies in this review highlighted several key genes, namely *CNTNAP2*, *GABRB3*, *JARID2*, *NLGN4X*, *NRXN1*, *PARK2*, *SHANK3*, and *UBE3A*, that appear to play significant roles in the ASD. These genes are involved in various neural development processes, including synaptic formation, neuronal connectivity, and brain signalling pathways. Understanding these genes and their interactions offers potential for new therapeutic approaches and better diagnostic tools. Here we provide a quick recap of the important findings of these genes related to ASD. 

### 4.1. CNTNAP2

Contactin-associated protein-like 2, encoded by the *CNTNAP2* gene, is a synaptic cell adhesion molecule (CAMs). *CNTNAP2* was pinpointed as a strong ASD risk gene by Baig et al. through in silico analysis of the relationship between genetic variants and protein structures [28]. The study illustrated that variants in *CNTNAP2* can distort neuron–neuron interactions that are crucial for signal transmission across the synaptic gap. *CNTNAP2* mutation altered the protein’s ability to bind with its synaptic partners, thereby impairing synaptic stability and plasticity. These distortions can affect critical areas of the brain, including language processing, cognitive function, and social behaviour, which are domains typically impacted in ASD [28]. Additionally, variants of *CNTNAP2* trigger an abnormal activation of activating transcription factor 6 (*ATF6*), a marker of endoplasmic reticulum (ER) stress. The activation of the ER stress response has significant implications for neuronal function and is linked to the developmental and functional abnormalities observed in ASD [29]. Findings of another study indicate that common variants in *CNTNAP2* are associated with differences in the connectivity of specific brain regions involved in language processing and sensory integration. The study further provides evidence that the impact of *CNTNAP2* on brain connectivity and multisensory integration might contribute to the communication difficulties observed in ASD [30].

### 4.2. GABRB3

γ-aminobutyric acid receptor subunit β-3 (*GABRB3*) is a subunit of the γ-aminobutyric acid (GABA) receptor, which conducts inhibitory signals in the central nervous system. Studies demonstrated that variants in the *GABRB3* gene are significantly associated with Asperger syndrome, a subtype of ASD. Moreover, these genetic variations are linked to a range of autism-related endophenotypes, such as sensory processing difficulties, issues with social cognition, and other cognitive challenges that are frequently observed in individuals with ASD [31]. The study also suggests that these associations may be due to the role of *GABRB3* in modulating inhibitory neurotransmission, which affects neural connectivity and the functional organisation of the brain [31]. Findings from other studies indicate that variants in the *GABRB3* gene are significantly associated with an increased risk of ASD by altering GABAergic signalling [32,33]. As GABA is a typical target in neurological diseases, diuretic bumetanide was investigated as a treatment of ASD [34].

### 4.3. JARID2

Jumanji, AT rich interactive domain 2 (*JARID2*) is located within chromosome 6p22.3. The gene product, Jumanji, is involved in the regulation of chromatin structure and gene expression through its interaction with the Polycomb repressive complex 2 (*PRC2*). Deletion of the region 6p22.3-p24.3, which affects not only *JARID2* but also neighbouring genes, suggested a complex interaction between multiple genes, including *ATXN1*, that contribute to the risk of ASD [35]. In a study of 16 patients with deletion of, or SNPs within, the *JARID2* gene alone, 16% were diagnosed with ASD, while all showed developmental delay [36]. The GWAS analysis conducted in another study pinpointed specific loci within the *JARID2* gene that were significantly associated with ASD in the East Asian cohort [37]. This finding suggests that *JARID2* may exhibit a unique presentation in certain populations that was not revealed in other studies using a Caucasian population dataset. 

### 4.4. NLGN4X

Neuroligin 4 X-linked (*NLGN4X*) encodes a protein that is a family member of neuroligins (NLGNs), which binds to neurexins (NRXNs) for the correct formation and maintenance of synapses in the brain [38]. Disruptions of the NLGNs-NRXNs interactions, caused by genetic alterations in *NLGN4X*, were implicated in the development of ASD [39]. In contrast, another study found no significant association between variants in the *NLGN4X* gene and the prevalence of ASD in the examined Chinese cohort [40]. The study provided several reasons why these findings might differ from other studies, including population genetic differences, sample size, and the specific genetic variants analysed. The absence of an association in this study indicates the complexity of ASD’s genetic interplay and suggests that the contributions of specific genes to the disorder may vary across different ethnic and genetic backgrounds [40].

### 4.5. NRXN1

The SHANK3-NLGN4-NRXN1 axis is a well-documented cascade responsible for synapse physiology. NRXNs, at the presynaptic side, and NLGNs, at the postsynaptic side, interact with each other to form functional synapses [41]. Mutation of the neurexins 1 gene (*NRXN1*) was firstly targeted by Feng et al. (2006) for its interaction with neuroligins (NLGNs). They confirmed the contribution of *NRXN1* to autism susceptibility [42]. In a large-scale comparative analysis involving over 1181 ASD families, hemizygous deletion of *NRXN1* was found to be associated with ASD [43]. *NRXN1* is located at chromosome 2p16.1. Kim et al. mapped breakpoints in this region in two ASD patients [44]. *NRXN1* was again identified as an ASD candidate gene by Glessner et al. using genome-wide high-resolution CNV detection [45]. More single nucleotide and structural variants of *NRXN1* were reported in ASD patients subsequently [46,47,48]. In a study of whole exon sequencing of 343 ASD families, *NRXN1* was revealed as the only one candidate gene for autism [49]. Interestingly, according to Wang et al., among the polymorphisms selected in their study, two SNPs were found associated with *NRXN2* and *NRXN3*, respectively, but not *NRXN1* in Chinese ASD patients [50]. Deletion of *NRXN1* was found to have strong association with autism, mental retardation, and language delay [51,52]. Taken together, *NRXN1* is regarded as one of the strong candidate genes for autism.

### 4.6. PARK2

*PARK2* (currently known as *PRKN*) encodes the E3 ubiquitin protein ligase Parkin, which is responsible for proteasomal degradation at the mitochondria. Mutation of the gene was heavily studied in Parkinson’s disease [53]. In the research of Dalla Vecchia et al., mutations or functional disruptions in *PARK2* were found contributing to neurodevelopmental disorders, including ASD, by affecting neuronal health and synaptic functioning [54]. Disruptions in *PARK2* were suggested to impact mitochondrial dysfunction and oxidative stress, which were hypothesised to play roles in the development of ASD [54]. Additionally, animal models harbouring *PARK2* mutations were shown to exhibit behavioural phenotypes resembling those presented in ASD, such as social deficits and repetitive behaviours [54]. In another study, *PARK2* was identified as a locus where rare CNVs were more frequently observed in individuals with ASD compared to control groups [55]. The study demonstrated that alterations in *PARK2* might lead to dysfunctional cellular mechanisms, particularly in neuronal cells, for which energy demand and protein regulation are critical for normal function [55]. Furthermore, this study underscores the importance of integrating different genetic approaches—such as examining both CNVs and single-nucleotide variations—to provide a more comprehensive understanding of the genetic factors contributing to ASD.

### 4.7. SHANK3

SH3 and multiple ankyrin repeat domains 3 (*SHANK3*), also known as *ProSAP2*, encodes a scaffolding protein at the excitatory synapses to enable proper positioning of neurotransmitter receptors [56]. *SHANK3* is located on chromosome 22q13. Deletion of the gene would result in Phelan–McDermid syndrome [57]. Durand et al. and Moessner et al. both reported the association of mutations and rare nonsynonymous variants of *SHANK3* with autism [58,59]. Later on, Schaaf et al. described *SHANK3* as one of the genes involved in the oligogenic heterozygous events carried by ASD patients [60]. In a meta-analysis, Leblond et al. revealed that copy number variants and truncating mutations in *SHANK* genes were present in 0.7% of the ASD patients [61]. Recently, Loureiro et al. also reported a recurrent frameshift variant of *SHANK3* in ASD patients [62]. *SHANK3* was also reported in one of our included articles having a significantly higher ratio of heterozygous deletion in Chinese patients [22]. On top of genetic risk factors, the *SHANK3* gene was extensively studied in neurobiology and in ASD disease pathology for its role in maintaining postsynaptic structure and hence synapse functioning [41,56].

### 4.8. UBE3A 

Ubiquitin protein ligase E3A (*UBE3A*) is one of the most extensively studied autism-linked candidate genes. It is located at 15q11-q13, a region found to have a high frequency of chromosomal anomalies in ASD patients [63,64,65]. The gene product of *UBE3A* is responsible for protein degradation in a cell and deletion of the gene causes Angelman syndrome [66]. In an early study by Cook et al., no linkage disequilibrium could be found for the gene *UBE3A* with autism, including the marker D15S122 [67]. Later on, Nurmi et al. observed a significant linkage disequilibrium (LD) with the same marker in autism patients [68]. This LD result was not replicable when other markers in the region were used in a dense linkage mapping study [69]. Concurrently, Veenstra-Vanderweele et al. identified several polymorphisms of the gene in 10 ASD patients. No functional mutation was detected [70]. Later, Glessner et al. reported in their genome-wide study that a CNV affecting *UBE3A* was found exclusively in ASD patients but not in controls using MPLA and qPCR for detection [45]. In the development of the ASD diagnostic tool, Bremer et al. revealed clearly the duplication of 15q11-q13, which covers *UBE3A*, in two patients [71]. Iossifov et al. identified a missense mutation in ASD patients that led to the hypothesis that the phosphorylation of *UBE3A* is important for the pathophysiology of autism [72,73]. There are a couple of genome-wide screening targeting structural variations associated with autism. *UBE3A* was not pinpointed in those studies [43]. Although molecular and cellular analyses shaped a putative mechanism, a concrete linkage between *UBE3A* and autism is yet to be revealed [74]. The challenges possibly lie with the complexity of the genome in that region. The fact that *UBE3A* is imprinted adds another layer of variety in symptom presentation.

This is the first systematic review of case-control studies focusing on SVs in ASD of Chinese ancestry. Compared with a trio study, case-control research has the advantage of excluding a similar genetic background that runs in a family. Interestingly, it was noticed that genetic background still remained as a challenge in some of the included case-control studies as ethnicity constraints were applied. Among the nine included articles, two of them raised the concern about the disparity of variant frequencies between Chinese and Caucasian. One of the major findings of Gazzellone et al. is that, in their dataset, the microduplication of *YWHAE* has a high frequency (0.9%) in Chinese population regardless of ASD condition, while they could not detect any of this event in their Caucasian control [20]. Similarly, Siu et al. observed a heterozygous variant of c.3388C>T and c.2204-14_2204-2dup at a frequency of 0.045 in their Southern Han Chinese healthy controls, compared with the allele frequency of c.3388C>T being 0.0059 in the 1000 Genomes Project [24]. Apart from these two included studies, Mak et al. also identified a CNV polymorphism enriched in the Chinese population in their autism genetic study [75]. A range of research addressed the effect of ethnicity on CNV [76,77,78]. In the study by Lou et al., thousands of Asian-specific CNVs were reported, while several hundreds of CNVs specific to Han Chinese were identified [77]. Park et al. also identified 3547 putative Asian-specific CNVs among the 5177 CNVs they detected in their study [76]. These findings reflected that ancestry-matched control can be of critical importance in analysing genetic risk factors of SVs. By this token, using a population of a different ancestry background as control in genetic analysis would increase the probability of mistakes in scientific results and may even lead to misdiagnosis if applied in clinical settings [79]. 

To overcome this situation, genome sequences of diverse populations are essential for more reliable scientific results and for more precise clinical diagnoses. A diverse genetic database enables researchers to identify ethnic-specific genetic markers [80]. It provides hints to the understanding of ethnic-dependent disease pathology and health disparities [81]. In addition to enriching the wide breadth of biomedical knowledge, the information is needed for more accurate diagnosis for patients of different ancestral backgrounds [79,82]. It enables effective translation into clinical practice and promotes personalized therapies [83,84]. The establishment of a global inclusive genetic database requires the efforts of different stakeholders in the society, including academic, clinical, and governance sectors. The basis of success relies on strategies that reduce bias, from inclusive recruitment of participants to proper and fair data sharing, to safeguard privacy and eliminate discrimination, among the many key factors in necessity [85].

In conclusion, the present review sheds light on the demand of a globally inclusive genetic database that permits universal studies of genetic diseases that are not biased towards specific genetic ancestries. This will have significant implications for policy, clinical practice, and research, ultimately leading to more personalised, equitable, and effective healthcare solutions for people worldwide. 

## 5. Limitations

This work reviews the extensive search of multiple databases and interpretation of results across different types of structural variations involving ASD. However, the limitation is that we were unable to complete a meta-analysis of SVs in ASD due to differences in study methodologies (e.g., selection of cases, diversion of participants) and analytical methods (e.g., sequencing thresholds, control for testing). 

## 6. Conclusions

To our knowledge, this is the first systematic review on case-control studies of SVs in ASD within the Chinese population. This review retrieved nine included studies, of which the quality was assessed by the STREGA checklist as low to moderate. It is revealed in the present work that specific SVs, including deletions, duplications, and copy number variations, are prevalent in the Chinese ASD cohort, affecting genes crucial for neural development and synaptic function. Some important genes for ASD were implicated in the included studies, namely *CNTNAP2*, *GABRB3*, *JARID2*, *NLGN4X*, *NRXN1*, *PARK2*, *SHANK3*, and *UBE3A*. This review enriches our understanding of the genetic composition of ASD among the Chinese population. Importantly, the essential role of ethnicity in genetic research was highlighted, implying the pressing need of a diverse genetic database across different ethnicities for future research and the development of unbiased, better healthcare systems globally. 

## Figures and Tables

**Table 1:** Summary of the characteristics of the included studies.

Study (Author/Year)	Study Settings	Participant Characteristics (Number/Diagnostic Method)	Methodology	Structural Variations Detected	Major Findings
Fan et al., 2018 [19]	Study design: Case-control Location: Shanghai Study period: Jul 2014–Dec 2017	Number of subjects: Proband: 401 Control: 197 ASD diagnostic method: DSM-5, ADOS and CARS Exclusion: Control group—major anomalies	CMA: Affymetrix CytoScan HD array with Chromosome Analysis Suite software Burden analysis: PLINK v1.07 and scripts developed in house	CNVs	The diagnostic yield of CMA was 4.2% (17/405 with clinically significant CNVs). Increased occurrence of rare loss events in the ASD cohort. Implied higher burden of rare gains in the severe ASD than the mild. Rare loss events disrupting genes extremely intolerant of LoF variants were found to be enriched in the ASD cohort. Rare CNVs in the *RIMS2* gene were found in two patients.
Gazzellone et al., 2014 [20]	Study design: Case-control Location: Harbin Study period: Jan 2007–Jun 2011.	Number of subjects: Proband: 104 Control: 875 (USA) Control: 1235 (China) ASD diagnostic method: DSM-IV, CARS and ABC Inclusion: Control—No developmental delay or autistic traits Exclusion: Subject—Rett syndrome, tuberous sclerosis, fragile-X syndrome, and any other neurological conditions suspected to be associated with autism were excluded by clinical examination and a molecular genetic test of the *FMR1* gene.	Affymetrix CytoScan HD array with Chromosome Analysis Suite software * PLANK confirm ethnicity	CNVs	Identified 241 rare CNVs in probands. Identification of the *YWHAE* CNV that appears to be a Chinese-specific polymorphism and not an ASD (or developmental delay)-associated variant. Uncovered nine de novo CNVs from eight probands. Uncovered four rare inherited CNVs from three probands.
Guo et al., 2017 [21]	Study design: Case-control Location: Changsha Study period: Not mentioned	Number of subjects: Proband: 406 ASD trios and 225 sporadic ASD cases Control: 1000 ASD diagnostic method: DSM-IV-TR Inclusion: ASD subjects diagnosed independently by two experienced psychiatrists. Control subjects had no history of ASDs or any other psychiatric diseases, nor did they have a familial history of psychiatric, neurological or autoimmune diseases.	Using Illumina HumanCNV370-Quad BeadChip and Illumina Human660W-Quad BeadChip. The CNVs were validated by quantitative PCR (qPCR).	CNVs	ASD patients had a significantly higher number of CNVs than the control subjects (odds ratio: 3.05, 95% CI: 1.66–5.74, *p* = 1.55 × 10^−4^, and Fisher’s exact test). A total of 32 rare CNVs were identified in 31 ASD probands (5.68%) and 19 CNVs were identified in 19 control subjects (1.92%). Among the 32 case-private CNVs, there were 16 de novo CNVs, 11 inherited CNVs, and 5 CNVs with unknown inheritance because of the absence of parental samples. Of 32 CNVs, five de novo duplications were found at 15q11–13. Compared the frequency of this CNV with primary European ancestry. The incidence of 15q11–13 duplications was significantly higher in the Chinese population in our study (5-fold, *p* = 0.021, and Fisher’s exact test). For de novo CNV, two deletions (3.5 Mb and 3.7 Mb) were found on chromosome Xp22.3, which includes the *NLGN4X* gene, in two ASD patients. Two deletions (1.39 Mb and 1.35 Mb) were found in 15q13.1–13.2, which includes the *APBA2* gene. Two duplications (1.9 Mb and 2.4 Mb) were detected on chromosome 3p26. Both duplications disrupted the gene encoding Contactin, *CNTN4*. Two duplications (1.1 Mb and 1.5 Mb) were detected on 2p12 in 2 ASD patients. No known gene was found in this region. For case-private CNVs, disruptions in four known ASD risk genes, *ARID1B*, *SHANK3*, *CDH10*, and *CSMD1* were found. One large de novo deletion at 6q24.3–6q27 contains the *ARID1B* gene, and one de novo deletion at 22q13.3 includes *SHANK3*. Two duplications disrupted genes encoding *CUB* and sushi domain-containing proteins (*CSMDs*). A maternally inherited deletion (2 Mb) at 5p14.2–14.1 disrupted a single gene, *CDH10*. *GRAMD2*, *CDH10*, and *STAM* were identified as novel potential ASD risk genes.
Liu et al., 2011 [22]	Study design: Case-control Location: Guangzhou Study period: 2009–2010	Number of subjects: *MLPA:* Proband: 75 Parents: 112 (non-autistic) Control: 30 *WGAA:* Proband: 6 Control: 6 ASD diagnostic method: DSM-IV and ABC Inclusion: Fulfill DSM-IV diagnosis criteria, ABC score above 53 Exclusion: Unclear diagnosis with other neurological disorders, serious physical illness, mental retardation, developmental language disorder, and other anomalies.	MLPA: SALSA MLPA kit P343 ABI PRISM 3100 Genetic Analyzer WGAA: Affymetrix Cytogenetics Whole-genome 2.7M Array SNP and variation suit 7 (Golden helix Co., Bozeman, MT, USA) Statistic: SPSS 11.0	Microdeletion CNVs	MLPA: - No abnormalities were found in 15q11-13, 15q13 microdeletion, and 16p11 microdeletion. - In 22q13, *SHANK3* gene loss of heterozygosity in exon 15 has a statistically higher frequency in cohort. WGAA: - CNV in chr 1,15,16,21,22 has a higher frequency in autistic patients, especially for chr.22.
Liu et al., 2012 [23]	Study design: Case-control Location: Changsha Study period: Not mentioned	Number of subjects: Proband: 313 Control: 500 ASD diagnostic method: DSM-IV-TR and CARS Exclusion: Subject—Fragile X syndrome Control—History of neurological disorders	Sequencing: ABI3100/3130 automated sequencer with the SeqMan program Analysis: Hardy Weinberg equilibrium—software SHEsis Statistics—SPSS 13.0	All kinds	Ten nonsynonymous missense variants, 7 missense variants, 3 deletions, and 12 synonymous variants (silence) were identified in the coding sequence and associated splicing regions of *NRXN1* in ASD patients. Identified the common SNP P300P of *NRXN1* significantly associated with risk of autism. All three deletions and four out of seven missense variants are not associated with ASD.
Siu et al., 2016 [24]	Study design: Case-control Location: Hong Kong Study period: Not mentioned	Number of subjects: Proband: 66 Control: 100 ASD diagnostic method: Adult group patients were diagnosed in childhood by psychiatrists, paediatricians or clinical psychologists before year 1990. The diagnoses were confirmed with the development, dimensional and diagnostic during adulthood. Paediatric group patients were diagnose by ADRI. Exclusion: Adult group—IQ score below 75 by WAIS-III	CGH: NimbleGen CGX-135K oligonucleotide arrays Sequencing: The GS Junior Benchtop System Mapping: GS Reference Mapper Evaluate variants: Polyphen-2 [16], SIFT, MutationTaster, HumanSplicing Finder and MaxEntScan	All kinds	One microdeletion of 1.97 Mb comprising 19 genes (incl. *NEO1*) was identified in an ASD patient. Five missense variants and one duplication were found in this region in other ASD patients. Defective nuclear translocation was shown in cultured cells carrying one of the *NEO1* mutations identified. One missense variant and one duplication was detected in a 10× higher frequency in southern Chinese.
Yin et al., 2016 [25]	Study design: Case-control Location: Taiwan Study period: Not mentioned	Number of subjects: Proband: 335 Control: 1093 Proband for replication: 301 Control for replication: 301 Diagnostic method: DSM-IV and confirmed by using the Chinese version of the Autism Diagnostic Interview-Revised (ADI-R). Exclusion: Subjects diagnosed as fragile X, Rett’s disorder, or other known chromosome/genetic disorders.	CNVs were called using Affymetrix Genotyping Console software v.4.1 (Affymetrix, Santa Clara, CA, USA). Genes overlapped with the CNV regions were reported according to UCSC genes (NCBI37/hg19. The case-specific CNVs were validated by SYBR-Green based genomic quantitative PCR (qPCR) using ABI StepOne Plus system	CNVs	Among the identified case-specific CNV loci, 17 were located in six chromosomal regions of the well-known ASD associated. CNV, 1q21.1, 15q11.2-13.1, 15q13.3, 16p11.2, 22q11.21, and 22q13.33. Among the genes overlapped with these CNV loci, only the *PARK2* gene was reported to be associated with ASD. Two exonic CNVs (one deletion, one duplication) were carried by two ASD cases while no exonic CNVs were detected in controls. We observed a fold change of 0.61 for exons 4–5, 0.48 for exons 6–7, and 0.58 for exons 9–11 in the proband and the father of Family U1984 when compared with unaffected controls (*p* = 0.014, *p* = 0.026, and *p* = 0.01, respectively). We observed a fold change of 0.46 for exons 3–4 and 0.56 for exons 4–5 in the proband and deletion-carriers of Family U2650 when compared with those of unaffected controls (*p* = 0.014 and *p* = 0.004, respectively). No significant difference in the expression level of exons 6–7 or exons 9–11 between controls and deletion carriers was found.
Zhao et al., 2020 [26]	Study design: Case-control Location: Shanghai Study period: Not mentioned	Number of subjects: Proband: 391 Control: 384 ASD diagnostic method: DSM-IV Inclusion: Diagnosis according to DSM-IV. Exclusion: Known mental and physical illness or chromosomal abnormalities.	High resolution melting (HRM) and Sanger sequencing	CNVs	Insertion deletions mutation (AACTC+/−) in the upstream 288 bp of CDS 2 were found in both case and control groups. The frequencies in the case and control groups were both 16/192 (0.083). There was no significant difference in the genotype frequencies between the case and control groups (*p* = 0.134).
Zhou et al., 2019 [27]	Study design: Case-control Location: Beijing and Tsingdao Study period: Not mentioned	Number of subjects: Proband: 539 Control: 512 ASD diagnostic method: ADI-R and ADOS Inclusion: Diagnosis according to DSM-IV. Inclusion: Case satisfy the same ADI-R criteria for ASD as used by the Simons Simplex Collection; a participant was used as a control if he/she had an AQ below 32, did not have any personal or family history of neurological disorders, psychiatric illness, or adverse pregnancy outcomes such as fetal loss, and had completed education through at least middle school to exclude any risk of low intellectual functioning.	A panel of 111 syndromic and 247 nonsydromic genes were selected and sequenced by Illumina Hiseq platform and validated by qPCR	CNVs	Seven de novo CNVs were found with targeted genes including *USH2A*, *SEMA5A*, *JARID2*, *PLXNA4*, *CNTNAP2*, *COMT*, *GNB1L*, and *TBX1*. Duplications of 15q1113 occurred in four cases, especially those in 15q13.3, which were found in three cases. The 15q13.3 duplications were found to be 375–514 kb, only encompassing *CHRNA7* and the first exon of *OTUD7A*.

Note. * For details, refer to the supplementary document of Gazzellone et al. (2014) [20].

**Table 2:** Reporting the quality of the included studies by Strengthening the Reporting of Genetic Association Studies (STREGA).

Item	Description	Fan et al., 2018 [19]	Gazzellone et al., 2014 [20]	Guo et al., 2017 [21]	Liu et al., 2011 [22]	Liu et al., 2012 [23]	Siu et al., 2016 [24]	Yin et al., 2016 [25]	Zhao et al., 2020 [26]	Zhou et al., 2019 [27]
1	Describe laboratory methods, including source and storage of DNA, genotyping methods, and platforms.	Yes	Yes	Yes	Yes	Yes	Yes	Yes	Yes	Yes
2	Describe any methods used to address multiple comparisons or to control risk of false positive findings.	No	Yes	Yes	No	No	No	No	No	Yes
3	Describe the centre at which the genotyping was performed.	No	Yes	No	No	No	No	No	No	No
4	Provide a hint on whether the genotyping was performed in one single batch or a few smaller batches.	No	No	No	No	No	No	No	No	No
5	Report the number of individual participants’ samples that were genotyped and how many of these samples were successfully genotyped.	No	Yes	Yes	No	No	No	Yes	Yes	Yes
6	Describe how to assess the level of and/or control for population stratification.	Yes	NA	NA	NA	NA	NA	NA	No	Yes
7	Describe any methods on determining the type of structural variations.	Yes	Yes	Yes	Yes	Yes	Yes	Yes	Yes	Yes
8	Stated whether this is the first report to report such genetic variations, was it a replicated effort of a previous study, or both.	No	Yes	Yes	No	Yes	Yes	No	No	No
Score (Max. 8)		3	6	5	2	3	3	3	3	5

Note. NA: Not applicable, because no population stratification was performed in the study.

**Table 3:** Summary of the structural variations reported in the included studies.

Chromosome Banding	SV	Size (kb)	Candidate Gene	Inheritance	Reference
1q21.1	Duplication	900	(Not reported)	(Not reported)	[19]
1p22.1-21.1	Deletion	12,232	56 genes (including *OLFM3*)	de novo	[21]
1q21.1	Deletion	1820	(Not reported)	(Not reported)	[19]
1q25.1	Deletion	689 (bp)	*RABGAP1L*	de novo	[25]
1p36.13	Duplication	33	*CROCC*	de novo	[25]
1p36.21	Duplication	161	*PRAMEF8*, *PRAMEF9*, *PRAMEF13*, *PRAMEF19*, *PRAMEF16*, *PRAMEF20*	de novo	[25]
1q41	Duplication	32	*USH2A*	de novo	[27]
2p11.2-p11.1	Duplication	335	*LOC654342*, *Mir_544*, *GGT8P*, *ACTR3BP2*	de novo	[25]
2p12	Duplication	1125	2 genes (including *CNTN4*)	unknown	[21]
2p12	Duplication	1500	(Not reported)	Paternal	[21]
2q12.2-12.3	Duplication	1544	5 genes (including *ST6GAL2*)	Paternal	[21]
2p16.3	Deletion	3 (bp)	*NRXN1*	(Not reported)	[23]
2p16.3	Deletion	12 (bp)	*NRXN1*	Paternal and Maternal	[23]
2p16.3	Deletion	15 (bp)	*NRXN1*	Paternal and Maternal	[23]
2p16.3	Deletion	19 (bp)	*NRXN1*	(Not reported)	[23]
2q36.3	Duplication	1072	*NYAP2*	Paternal	[21]
2q37.1	Deletion	22	*GIGYF2*	de novo	[20]
3p12.3	Duplication	291	*MIR1324*, *FLJ20518*, *LOC401074*, *ZNF717*, *MIR4273*	de novo	[25]
3p14.1	Duplication	3207	(Not reported)	(Not reported)	[19]
3q22.1	Duplication/Deletion	108	*ALG1L2*, *FAM86HP*	de novo	[25]
3p26.3	Duplication	1859	10 genes (including *CNTN4*)	unknown	[21]
3p26.3-26.2	Duplication	2402	2 genes (including *CSMD1*)	unknown	[21]
4q13.2	Deletion	39	*UGT2B15*, *UGT2B17*	de novo	[25]
4p16.1	Duplication/Deletion	258	*MIR548I2*, *AB059369*	de novo	[25]
4p16.3	Duplication	305	*DQ584669*, *FAM86EP*, *BC042823*, *OTOP1*	de novo	[25]
4q22.2	Deletion	28	*GRID2*	Maternal	[20]
4q28.1	Duplication	982	*SPRY1*, *SPATA5*	de novo	[20]
4q31.21q33	Duplication	25,264	(Not reported)	(Not reported)	[19]
4q35.2	Deletion	1004	*LINC01060*	Paternal	[21]
5q13.2	Duplication	1631	12 genes	Maternal	[21]
5p14.2-14.1	Deletion	2021	*CDH10*	Maternal	[21]
5p15.31	Duplication	62	*SEMA5A*	de novo	[27]
5p15.33-15.2	Deletion	8642	55 genes (including *SLC9A3*)	de novo	[21]
5q35.3	Duplication	1070	11 genes (including *GRM6*)	Paternal	[21]
6p22.3	Duplication	36	*JARID2*	de novo	[27]
6q24.3-q27	Duplication	21,867	126 genes (including *ARID1B*)	de novo	[21]
6q26	Duplication/Deletion	493	*PARK2*	de novo	[25]
7q11.23	Deletion	1512	(Not reported)	(Not reported)	[19]
7q32.3	Duplication	39	*PLXNA4*	de novo	[27]
7q35	Duplication	726	*CNTNAP2*	de novo	[27]
8p23.1	Duplication	45	*LONRF1*, *MIR3926-1*, *MIR3926-2*	de novo	[25]
8q23.3	Duplication	1802	2 genes (including *CSMD3*)	Maternal	[21]
8p23.3-p22	Deletion	15,273	102 genes (including *RP1L1/XKR6*)	de novo	[21]
8p23.3p23.1	Deletion	9979	(Not reported)	(Not reported)	[19]
8p23.3-23.2	Duplication	1064	7 genes (including *STAM*)	unknown	[21]
9p21.1	Deletion	139	*LINGO2* (intronic)	Paternal	[20]
9p21.1	Deletion	132	*LINGO2*	Maternal	[20]
9q13	Duplication	507	*AK308561*, *BC080605*, *LOC642236*	de novo	[25]
10p12.33	Deletion	73	*SLC39A12*	Paternal	[20]
10q11.2	Duplication	5606	(Not reported)	(Not reported)	[19]
10q12.33	Duplication	1103	(Not reported)	unknown	[21]
12p13.31	Duplication	158	*LINC00937*, *FAM86FP*, *FAM90A1*	de novo	[25]
14q11.2	Duplication/Deletion	84	*DHRS4*, *DHRS4L2*, *DHRS4L1*	de novo	[25]
15q11q13	Duplication	5775	(Not reported)	(Not reported)	[19]
15q11q13	Duplication	5250	(Not reported)	(Not reported)	[19]
15q11q13	Duplication	5790	(Not reported)	(Not reported)	[19]
15q11.2-q13.1	Duplication	5913	119 gene (including *UBE3A*, *GABRB3*)	de novo	[21]
15q11.2-q13.1	Duplication	5894	109 gene (including *UBE3A*, *GABRB3*)	de novo	[21]
15q11.2-q13.3	Duplication	10,923	162 gene (including *UBE3A*, *GABRB3*)	de novo	[21]
15q11.2-q13.3	Duplication	10,450	155 gene (including *UBE3A*, *GABRB3*)	de novo	[21]
15q13.1-13.2	Deletion	1391	6 genes (including *APBA2*)	de novo	[21]
15q14	Duplication	1277	19 genes	Maternal	[21]
15q23	Deletion	4320	34 (including *GRAMD2*)	de novo	[21]
15q23-24.1	Deletion	1969	19 genes (including *NEO1*)	(Not reported)	[24]
15q24.1	Duplication	13 (bp)	*NEO1* (intronic)	(Not reported)	[24]
16p11.2	Duplication	1150	6 genes	de novo	[21]
16p11.2	Deletion	232	9 genes	de novo	[20]
16p11.2	Deletion	598	(Not reported)	(Not reported)	[19]
16p13.3	Duplication	319	7 genes	de novo	[20]
16p13.3	Duplication	327	15 genes	de novo	[20]
16p13.11	Deletion	845	(Not reported)	(Not reported)	[19]
17p12	Deletion	1397	(Not reported)	(Not reported)	[19]
17p12	Deletion	1404	(Not reported)	(Not reported)	[19]
17p12	Deletion	1340	7 genes	Maternal	[21]
17p13.3-p13.2	Duplication	994	16 genes	de novo	[20]
19q13.42	Duplication	1357	>60 genes	de novo	[25]
20q13.31-13.33	Duplication	6760	96 genes	de novo	[21]
21q11.2	Duplication/Deletion	138	*ANKRD30BP2*	de novo	[25]
22q11.2	Deletion	1254	(Not reported)	(Not reported)	[19]
22q11.2	Deletion	2882	(Not reported)	(Not reported)	[19]
22q11.21	Deletion	215	*COMT*, *GNB1L*, *TBX1*	de novo	[27]
22q11.23	Deletion	8	*GSTTP2*	de novo	[25]
22q13	Deletion	(Not reported)	*SHANK3*	de novo/Maternal	[22]
22q13.31-13.33	Deletion	2627	40 genes (including *SHANK3*)	de novo	[21]
Xq13.2	Deletion	33	*NAP1L6*	de novo	[20]
Xp21.1	Deletion	154	*DMD*	de novo	[20]
Xp21.1	Deletion	55	*DMD*	de novo	[20]
Xp22.33-22.31	Deletion	3536	6 genes (including *NLGN4X*)	de novo	[21]
Xp22.32-22.31	Deletion	3695	10 genes (including *NLGN4X*)	de novo	[21]

**Table 4:** Summary of the genes reported in this review.

Candidate Gene	Chromosome Banding	SV	Inheritance	Reference
*CNTNAP2*	7q35	Duplication	de novo	[27]
*GABRB3*	15q11.2q13.1; 15q11.2-q13.3	Duplication	de novo	[21]
*JARID2*	6p22.3	Duplication	de novo	[27]
*NLGN4X*	Xp22.33-22.31; Xp22.32-22.31	Deletion	de novo	[21]
*NRXN1*	2p16.3	Deletion	Paternal and Maternal	[23]
*PARK2*	6q26	Duplication/Deletion	de novo	[25]
*SHANK3*	22q13.31; 22q13.31-13.33	Deletion	de novo/Maternal	[21,22]
*UBE3A*	15q11.2-q13.1; 15q11.2-q13.3	Duplication	de novo	[21]
